# Comparing the Role of Mechanical Forces in Vascular and Valvular Calcification Progression

**DOI:** 10.3389/fcvm.2018.00197

**Published:** 2019-01-10

**Authors:** Madeleine A. Gomel, Romi Lee, K. Jane Grande-Allen

**Affiliations:** Department of Bioengineering, Rice University, Houston, TX, United States

**Keywords:** valvular calcification, vascular calcification, biomechanics, CAVD, atherosclerosis, oscillatory stress, shear stress

## Abstract

Calcification is a prevalent disease in most fully developed countries and is predominantly observed in heart valves and nearby vasculature. Calcification of either tissue leads to deterioration and, ultimately, failure causing poor quality of life and decreased overall life expectancy in patients. In valves, calcification presents as Calcific Aortic Valve Disease (CAVD), in which the aortic valve becomes stenotic when calcific nodules form within the leaflets. The initiation and progression of these calcific nodules is strongly influenced by the varied mechanical forces on the valve. In turn, the addition of calcific nodules creates localized disturbances in the tissue biomechanics, which affects extracellular matrix (ECM) production and cellular activation. In vasculature, atherosclerosis is the most common occurrence of calcification. Atherosclerosis exhibits as calcific plaque formation that forms in juxtaposition to areas of low blood shear stresses. Research in these two manifestations of calcification remain separated, although many similarities persist. Both diseases show that the endothelial layer and its regulation of nitric oxide is crucial to calcification progression. Further, there are similarities between vascular smooth muscle cells and valvular interstitial cells in terms of their roles in ECM overproduction. This review summarizes valvular and vascular tissue in terms of their basic anatomy, their cellular and ECM components and mechanical forces. Calcification is then examined in both tissues in terms of disease prediction, progression, and treatment. Highlighting the similarities and differences between these areas will help target further research toward disease treatment.

## Introduction

In the cardiovascular system, the heart works with surrounding vasculature to pump blood throughout the body. While the heart valves and vasculature are different in gross morphology, cellular structure, and in the forces they experience in the body, they both calcify and become fibrotic due to disease. Calcification is the irregular deposition of mineralized crystals that change both the micro- and macro-scale properties of tissue. It is a complex, ill-defined disease despite continuous investigation and is still being actively investigated. Although both heart valves and vasculature exhibit calcification and share some of the underlying processes leading toward mineralization, few significant correlations between the two have been made. In fact, while these conditions are seemingly similar, treatments that help reduce calcification in vasculature have been shown to have no effect on valvular calcification. As such, most research has looked at vascular and valvular calcification separately although they share similar risk factors and may have overarching parallels. Understanding the key differences and similarities between these two anatomic areas where calcification occurs may guide research efforts toward better treatments for both.

In valves, calcification is present as Calcific Aortic Valve Disease (CAVD). CAVD is the mineralization of heart valves that includes nodule formation and stenosis—the narrowing of the valve opening (1). CAVD begins as a mild sclerosis of the valve that worsens to stenosis and late-stage calcification. Of the adult population older than 75 years old, 2.8% are reported to have either moderate or severe calcific aortic stenosis (2). The calcification of valves was once thought to be a passive degradation over time, although multitudes of studies now prove that it is an active and highly regulated process that involves biochemical signaling, mechanical stimuli and cellular responses (3, 4). Calcification of aortic valves starts on a nanoscale level with calcific nodule formation. These nano-nodules coalesce to form larger micro- and macro-scale nodules. Currently, there is no treatment for valvular calcification and most patients do not experience noticeable symptoms until the disease has progressed substantially. When the disease is highly advanced, valvular replacements are required to restore healthy physiology. As the aortic valve controls the laminar flow of blood into the vascular system, disturbed flow from calcified valves can have downstream effects on the vascular system (5).

Vascular calcification is commonly observed as atherosclerosis or as a downstream secondary effect of a different disease. A majority of individuals over the age of 60 have calcification exhibiting as enlarging calcium deposits found in their arteries (6). Similar to CAVD, vascular calcification was initially linked to natural processes associated with aging (7), but is now understood to be a complex disease in need of further study. Common conditions such as diabetes, osteoporosis, kidney failure, and menopause are associated with vascular calcification (8, 9). Furthermore, vascular calcification can be a downstream effect of valvular calcification due to the changes in the mechanical environment brought on by the malformed calcified valves (6, 10, 11).

Due to the extreme prevalence of calcification in our society, and the lack of nonsurgical interventions for valvular calcification, there is a need to fully understand these two diseases, their similarities and their differences. Understanding the key differences between vascular and valvular calcification along with the role of mechanical force in their progression will help pinpoint strategies for highly specific treatment options.

## Cardiovascular Structure and Function

### Valvular

The heart is comprised of four chambers that are separated from one other and from nearby vasculature by four valves. Out of the tricuspid, mitral, pulmonary and aortic valves, the aortic valve is often the source of the most clinically serious calcification and will therefore be the valve addressed in this review.

**Figure 1:**
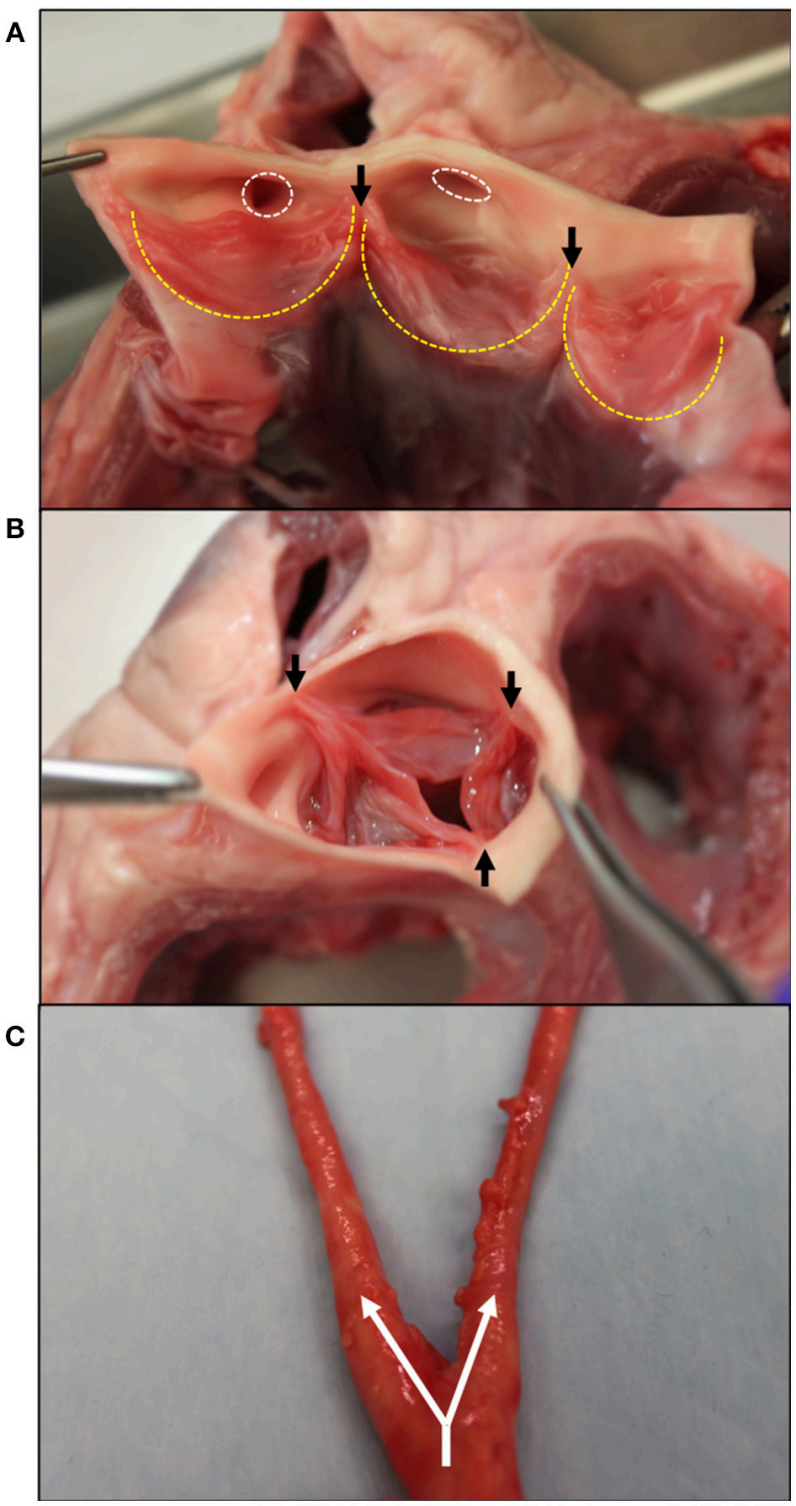
Gross morphology of the aortic valve and branching carotid artery. **(A)** Bisected aortic valve opened to show the three leaflets (outlined in yellow). On the left, the two coronary leaflets can be seen with the coronary outflows circled in white superior to the leaflets. **(B)** An intact aortic valve shows the leaflets coapting, starting at the commissure edges (black arrows). **(C)** The carotid artery at its bifurcation–the separation of one large artery into multiple smaller arteries. Shown with white arrows, the common carotid splits into the internal and external carotids. Tissue obtained from young adult (6–9 month) porcine specimens (Animal Technologies, Tyler, TX).

The aortic valve (AV) separates the left ventricle of the heart from the aorta and is responsible for maintaining the unidirectional flow of oxygenated blood. Three semilunar leaflets form the AV and connect at the aortic root inferior to the sinuses of Valsalva (Figure 1A). The three leaflets meet at junctions called commissures (Figure 1B).

As seen in Figure 1A, two of these leaflets have superior outflows to the coronary arteries and are thus referred to as the left and right coronary leaflets (5). The remaining leaflet is denoted as the non-coronary leaflet.

**Figure 2:**
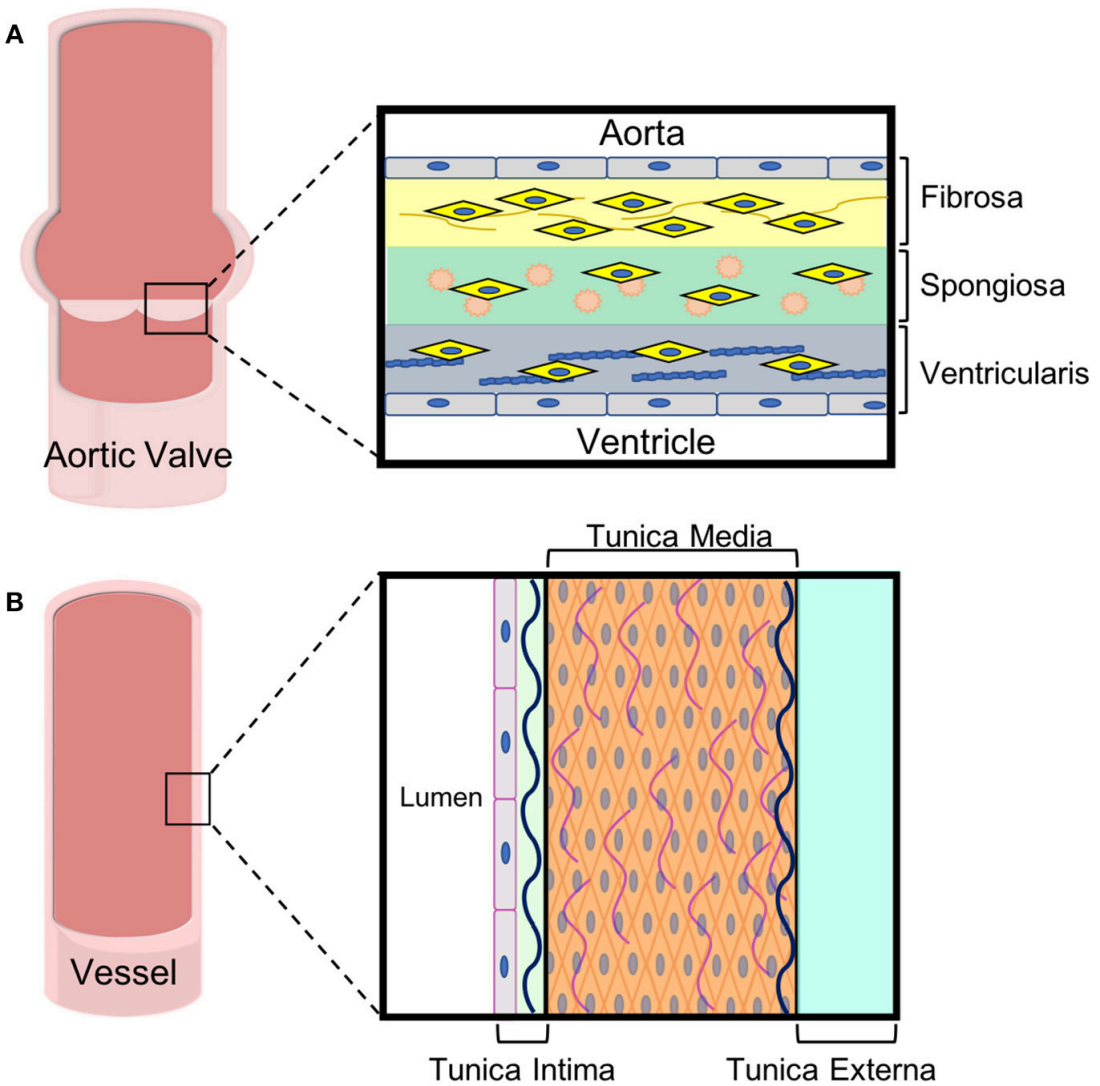
ECM and cell composition of aortic leaflets and vascular walls. Both **(A)** aortic valve leaflets and **(B)** vessel walls are tri-laminar structures with specific cell and extracellular matrix in each layer. These specialized layers impart different mechanical properties to the tissues, which are important for their functions.

In adults, the AV leaflets are avascular with a tri-layered structure of cells and extracellular matrix (ECM), which is essential to the overall mechanics of the valve (12). The layers of the AV leaflets are denoted based on their orientation to the ventricle and aorta (13). The layer closest to the ventricle is the ventricularis and is mostly comprised of elastin fibers (Figure 2A). The ventricularis is lined on its outer surface with valvular endothelial cells (VECs) and contains valvular interstitial cells (VICs). On the aortic side, the fibrosa layer has a similar orientation of cells to the ventricularis with VECs lining the outside of the valve and VICs in the interior but consists of ECM rich in collagen fibers. Between these layers, there is the spongiosa, which is populated by VICs surrounded by ECM laden with proteoglycans and glycosaminoglycans (GAGs).

The varying ECMs of AV leaflets impart differing mechanical properties to each of the leaflet layers (12). Elastin gives the ventricularis layer elastic recoil, which is important for the repeat flexing of this layer during the cardiac cycle. The fibrosa layer contains crimped and circumferentially-oriented collagen that gives valve tissue its unique anisotropic and nonlinear stress-strain response and its strength (14–18). In terms of dry weight, the whole valve leaflets are comprised mostly of collagen type I and III (19, 20) with type I being more abundant (21). The spongiosa acts like an intermediary between the ventricularis and fibrosa, dispersing the strong forces felt by both and cushioning the overall effect (18). Hyaluronan is a series of repeating disaccharides that forms the majority of the spongiosa. Hyaluronan's negative charge attracts water molecules, which gives the spongiosa its unique mechanical properties (19, 22, 23).

As mentioned previously, the AV leaflets consist primarily of two cell types: VICs and VECs. VECs line the surface of the valve leaflets on the ventricularis and fibrosa sides. These cells directly interact with blood and the shear forces associated with its flow through the valve. VECs are mechano-sensitive cells that regulate valve hemostasis based on the mechanical forces they experience (24). VECs help to maintain physiological balances between the valve and its environment but are often considered one of the first mechanisms in valve calcification. These cells can go through an Endothelial to Mesenchymal Transition (EndoMT), which can cascade down to an osteogenic genotype and initiate calcification (25, 26).

VICs are located throughout the three layers of the AV leaflets, but are more populous in the fibrosa layer (27). VICs have multiple phenotypes that can be found in the valve (28–31). Quiescent VICs (qVICs) are the most abundant in healthy adult valve tissue. qVICs can transition to activated VICs (aVICs) through changes in transforming growth factor and smooth muscle alpha actin levels (32). During fetal development, aVICs are responsible for initially producing ECM and creating the valve (33). After birth and throughout childhood, the number of aVICs decreases rapidly. In adults, aVICs are observed when a stress or trauma incite the cells to transition from qVICs to aVICs. VICs sense these traumas through their innate mechano-sensitivity, which allows them to respond to changes in the mechanical environment. aVICs can further differentiate into osteoblastic VICs (oVICs), which produce higher levels of alkaline phosphatase and can lead to calcification (34–36). In healthy valves, the interaction between VICs and VECs is an important deregulator in calcification, producing anti-fibrotic factors that reduce the abundance of oVICs (37). It has also been shown that VICs can decrease the propensity of cells going through EndoMT to become osteoblastic (26).

### Vasculature

The vascular system works to carry blood and lymph throughout the body. Arteries carry oxygenated blood away from the heart to the organ systems, while veins return deoxygenated blood to the heart and lungs. Capillaries are small blood vessels that aid in the exchange of oxygen between blood and surrounding tissue throughout the body and in the lungs. Most arteries and veins branch from larger vessels (Figure 1C). Arteries and veins consists of three layers: an endothelium, smooth muscle cells, and connective tissue (38). The connective tissue found in vasculature mainly consists of elastin and collagen, which determine the tissue's mechanical properties. While the proportion of collagen to elastin in the vessel wall changes through the body, together they usually account for roughly 60% of the vessel's dry weight (39). Similar to their roles in valves, elastin provides elastic recoil, extensibility and load bearing to the vasculature whereas collagen also affects tissue strength and extensibility.

The innermost vessel layer, the *tunica intima*, is the thinnest and is comprised of a single layer of flat endothelial cells, and a polysaccharide ECM (Figure 2B). The *tunica media*, the middle layer, consists of concentric elastic fibers, circumferentially aligned collagen, and vascular smooth muscle cells (39, 40). The vascular smooth muscle cells (VSMCs) are largely responsible for the ability of vasculature to carry blood and lymph throughout the body. In arteries, the *tunica media* makes up the thickest layer, about 50% of the dry weight, as greater musculature is needed to distribute blood throughout the entire body. The outermost layer is the *tunica externa*, which is made entirely of collagen and elastin. The *tunica externa* is the thickest layer in veins. In both arteries and veins, the *tunica externa* is also the primary location of the vasa vasorum, the network of capillaries supplying the cells within thick-walled blood vessels with oxygen and nutrients. The capillaries of the vasa are mainly comprised of endothelial cells.

The contraction and relaxation of the VSMCs in the *tunica media* change the physical volume within the blood vessels and have the ability to control local blood pressure. Many vessel-related pathological conditions can be traced back to problems stemming from VSMCs and their responses to mechanical stimulation. Issues regarding hypertension are due to higher levels of vasoconstriction by these smooth muscle cells (41). Furthermore, plaque formation, inflammation, and atherosclerosis, which accompany calcification, are heavily driven by the excessive proliferation of vascular smooth muscle cells (42).

### Comparison of Anatomy and Composition

Both valves and vasculature are important regulators in the movement of blood throughout the body. Valves and vasculature are both comprised of a specific, layered structure of cells and ECM that defines their mechanical attributes. While both consist of elastin and collagen, the variation in ECM densities between the two tissues shows the variation in the mechanical stimuli to which they respond. Vascular anatomy is dominated by elastin, which allows the tissue to respond elastically to varying blood pressures. The aortic valve has a tri-layered structure with more collagen than elastin owing to the demanding need of mechanical strength while still maintaining elastic recoil. The endothelial cells present at the blood interfaces of these tissues are essential to both vascular and valvular health and homeostasis. The cellular components found within the interior of valves and vasculature are vastly different and are likely key contributors to their pathologies as described later.

## Physiological Mechanical Forces

### Valvular Forces

**Figure 3:**
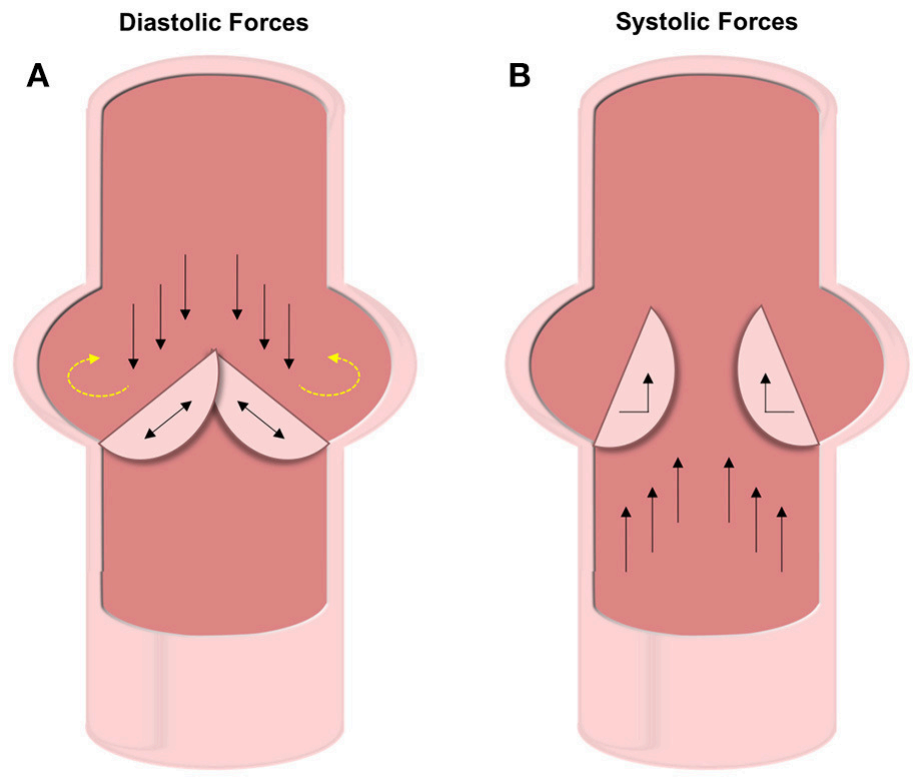
Diastolic and systolic forces on the valve leaflets. **(A)** Forces experienced during diastole include compression and oscillatory shear on the fibrosa VECs and tensile strain on the VICs. The oscillatory forces (shown with yellow dashed arrows) are thought to be the cause of initial calcification. **(B)** Under systolic forces, the ventricularis VECs experience straight shear while the VICs feel bending forces.

The aortic valve experiences roughly 3 billion cycles of opening and closing during the average human lifespan (43). The cardiac cycle involves both systole and diastole, which impart different forces on the AV leaflets (12, 44). During systole, blood pumps from the ventricle out to the aorta causing the AV leaflets to separate and bend toward the sinuses of Valsalva (Figure 3B). Three forces are felt during this stage of the cardiac cycle: VICs experience bending strain in the leaflets at the annulus (the line of attachment to the aortic root), and VECs experience both laminar shear against the ventricularis as blood is ejected from the ventricle and oscillatory shear in-between the fibrosa and the aortic wall.

As the valve closes for the ventricle to be refilled during diastole, the AV leaflets coapt under axial pressure, generating a tensile strain along the length of the leaflets in the VICs (Figure 3A). The fibrosa side of the AV leaflets undergo significant oscillatory forces due to the filling of the sinuses of Valsalva (13). During this backfilling, the coronary and non-coronary leaflets experience differing forces. The coronary leaflets have arterial outflows where blood flows through straight shear and therefore reduces the oscillatory nature of the hemodynamic force. However, on the non-coronary leaflet there is no outlet and thus the blood pools and exerts significant oscillatory shear forces.

Over the full cardiac cycle, the ventricularis experiences higher and unidirectional forces due to blood flow, while the fibrosa has lower wall shear stresses (WSS) that are bidirectional—especially in the non-coronary leaflet (45). Findings by Cao et al. also show that WSS varies across the leaflet orientation, with radial WSS being significantly a higher component to the total WSS than is circumferential. As discussed later, these variations in forces experienced by different locations in the AV play a large role in the initiation and progression of calcification.

### Vascular Forces

**Figure 4:**
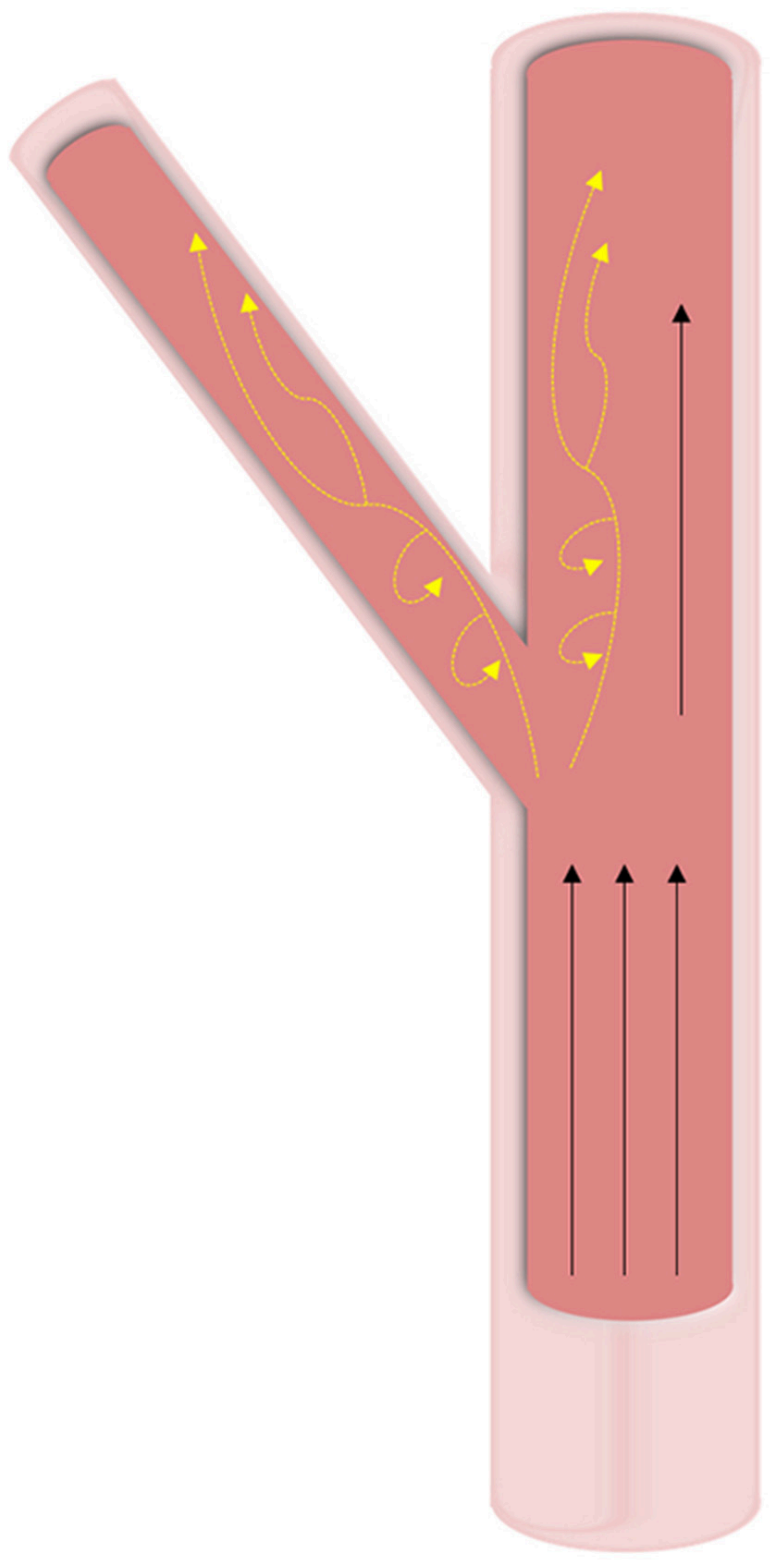
Hemodynamic forces in vasculature. Wall shear stresses in vasculature tissue is highly dependent on geometry. At straight sections, high unidirectional shear forces are predominant. At bifurcations and curved regions, oscillatory shear (shown with yellow dashed arrows) is experienced at lower pressures and leads to calcification.

In vasculature, the force of blood flow within the vessels—pulsatile endothelial shear stress—is the most significant and prominent. The intensity of shear stress faced by vasculature differs based on both the shape of the vessel and the location of the vessel within the body (Figure 4). Straight regions of arterial trees face laminar blood flow that provides high and constant pressure (>15 dyne/cm^2^). Vascular regions that branch and curve experience non-uniform, irregular, and disturbed blood flow. Due to the variability in blood flow in these curved regions, the WSS applied to the vascular endothelial cells is noted to be much lower than in straight regions (<4 dyne/cm^2^) (46, 47). Bifurcations in arterial trees can show low shear stress and are the greatest targets for diseases like calcification, among other clinical conditions (48). Due to the low shear stress and the recirculation of blood in the oscillatory flow, blood components have more time to interact with the vascular wall leading to pathologies.

In healthy vasculature experiencing high and constant WSS, molecules responsible for anti-inflammatory, anti-thrombotic, anti-apoptotic properties and vasodilation are consistently expressed and upregulated while molecules known for inflammatory responses are downregulated. One of the many molecules controlled by shear stress is nitric oxide (NO). Similar to its role in valves, NO is necessary for healthy vasculature due to its anti-inflammatory properties (49). High shear stress serves as a continuous stimulus for the endothelium to produce NO and prevent pathological changes such as calcification and atherosclerosis. High shear stress also controls the migration, differentiation, and proliferation of VSMCs. Healthy vasculature with high pressure ensures that the VSMCs remain in the *tunica media* and don't proliferate in the *intima*. VSMCs in the *intima* produce fibrillar collagen, which, over time, contribute to atherosclerotic plaque (46, 47).

### Comparison of Mechanobiology

In summary, straight segments of the vasculature are exposed to mostly laminar shear stresses at a near consistent pressure. At branches in the vasculature and directly downstream of the branches, these stresses shift to become more oscillatory, lower magnitude shear stresses. Despite this change at the geometric variation, overall the majority of stresses are unidirectional and constant. In contrast, the aortic valve is under multiple types of stresses that are constantly changing due to the cyclical opening and closing of the valve. The mechanical forces experienced by the ventricularis and fibrosa sides of the valve leaflet differ, and the non-coronary leaflet experiences different forces than the coronary leaflets. Both the non-coronary leaflet and the regions of the vasculature distal to bifurcations experience lower, more oscillatory forces compared to the rest of the vasculature and the other valve leaflets, respectfully. These anatomic distinctions and associated stresses are shown to be a key factor in calcification discussed later in this review.

## Calcification of Cardiovascular Tissue

### Valvular Calcification

Clinically, mineralization of the aortic valve is observed in patients who are in the late stages of CAVD because patients tend not to seek treatment for mild and moderate cases as symptoms are not yet severe (50). Due to insensitive testing modalities, early calcification is also difficult to study *in vitro* as extremely low calcium levels are difficult to distinguish from background noise. Thus, the initiation of calcification either *in situ* or *in vitro* remains undetermined and debate on the exact mechanisms that initiate calcification in valves still persists. In fact, until recently calcification was considered a passive deterioration of the heart valve instead of the active disease it is now understood to be. While specifics are unclear, it is now well-accepted that there is a complex crosstalk between cells, ECM, biochemical cues and biomechanical changes during CAVD, and that these interactions drive mineralization.

**Figure 5:**
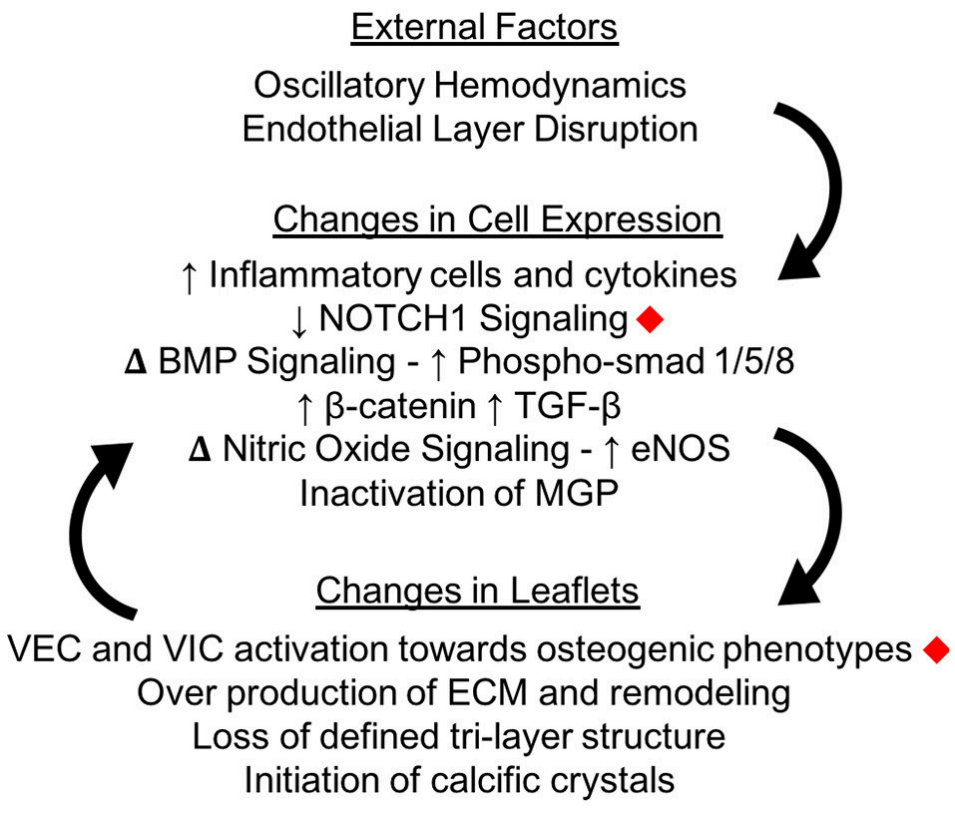
Calcification changes cell expression in the aortic valve. The oscillatory shear stresses experienced by VECs on the fibrosa side of the valve can lead to endothelial layer disruption as well as specific changes in cellular pathway expressions. Changes in cellular expression can change the macro structure of the valve leaflets, which creates a positive feedback loop instigating calcification. Changes marked with red diamonds have been expressly linked to mechanical shear stresses in the aortic valve.

Calcification in the aortic valve can appear as osteogenic nodules similar to that found in bone (51). When calcification starts, small scale crystals form within the AV leaflets (52). The initiation of these crystals is still being investigated. As described below, some studies propose that the process of activated VICs remodeling their surroundings causes these crystals, while others focus on endothelial disruption allowing calcium phosphate from the blood into the tissue. A combination of these factors is likely. Once initiated, calcification causes a positive feedback loop of mechanical changes, ECM remodeling and VIC activation which promote further calcification progression (Figure 5).

The progression of calcification follows a reliable pattern based on the mechanical environment of the leaflets and cells (53). Of the three leaflets, the non-coronary leaflet is the most likely to calcify (52, 54–56). This susceptibility is likely due to the changes between the oscillatory shear forces experienced by the non-coronary leaflet compared to the more laminar shear on the coronary leaflets as discussed earlier. VICs in the non-coronary leaflet have an increased expression of calcific markers, more osteogenic differentiation, and more mineralization compared to the coronary leaflets (56).

Individual leaflets also show expressional differences based on the different sides of the leaflet and the physical forces these sides experience. Ge et al. has shown through computational modeling that the endothelial cells on the fibrosa and ventricularis experience extremely varying shear forces (57). The fibrosa layer of AV leaflets is consistently proposed to be the initiation point for calcific nodules (58), since it is directly exposed to the bidirectional oscillatory shear forces at the valve/blood interface. This would also be consistent with the observation that oscillatory forces are highest for the non-coronary leaflet, therefore the fibrosa side that is in contact with these forces would be most affected by mineralization. Usually, calcification originates at the line of attachment where the leaflet meets the valve wall or in the belly region of the leaflet (52). Calcification continues to propagate in a radial manner that follows the lines of highest shear stress in the leaflets (59).

The changing mechanical environment of the cells is accompanied by disruption of the leaflet ECM. Collagen becomes more disorganized and distinguishing between the tri-layered ECM becomes more challenging (27, 60). As detailed earlier, the tri-layered structure is integral to the overall function of the AV. The hyper-physiological mechanical forces experienced by the valve in calcification negatively impacts the function of the spongiosa. While normally the spongiosa has minimal collagen when compared to the fibrosa, during calcification the spongiosa has significant collagen deposition due to aVICs (61). This change in ECM reflects the impaired mechanics, as the spongiosa normally works to absorb and dissipate the forces experienced by the other two layers but in CAVD the central layer loses this ability. The collagen deposition in this layer follows the progression of calcific nodule formation as it moves from the superficial fibrosa side throughout the leaflet.

At the micro-level, there are still many questions to be answered about the effects of mechanical stimulation on cellular pathway activation and inactivation. Specific pathways such as Wnt (62, 63), NOTCH (64), TGF-β (65), and BMP (66) have been shown to play an important role in calcification but little is known about how the mechanical environment of the valve affects most of these pathways (67).

Recent breakthroughs have revealed new understanding of the role of mechanical force in influencing these pathways. Pairing finite-element analysis with *in vitro* cell culture, Weinberg et al. showed that endothelial cells exposed to ventricularis-like stresses had higher levels of “atheroprotective” factors than endothelial cells under fibrosa-like stresses (68). NOTCH1, a transmembrane protein important in regulating endothelial cells, has been shown to be activated by shear stresses in aortic VECs (69). Oscillatory shear stresses on endothelial cells *in vitro* were shown to cause an upregulation of cells going through EndoMT as demonstrated by increased αSMA expression, and had higher levels of inflammation as shown by ICAM1 and NFKβ1 expression (70).

The calcific aberrations in AV leaflets are credited with increasing local stiffnesses of the leaflet. With this local stiffness increase and the disruption of the underlying ECM, both VECs and VICs vary their responses and drive the valve further toward calcification. VECs upregulate their production of inflammatory cytokines such as TGF-β, which makes VECs more likely to go through EndoMT (71). The disruption of the endothelial layer also allows for the infiltration of inflammatory cells into the AV leaflets. These inflammatory cells, which are predominantly macrophages, can play active roles in the progression of calcification and remodeling of surrounding ECM (72, 73).

Endothelial disruption can also affect the underlying mechanical properties of the valve, which can then in turn promote VIC differentiation (74). VECs have been shown to signal the activation and calcification of VICs through the regulation of NO (75, 76), an important inhibitor of calcification (77–79). The disruption of the endothelial layer also allows for an influx of inflammatory cells into the cell as well as an upregulation of osteogenic mediators (potential drug targets).

VICs have been widely reported to transition to an activated state when their relative ECM becomes stiffer (80, 81). For both aVICs and VECs, the process of EndoMT can hasten their transition toward an osteogenic phenotype, creating larger nodules of calcification. Thus, the cycle of calcification continues as the mechanical properties of the valve progress further from physiological, as ECM changes further, and as more cells become activated (82).

**Figure 6:**
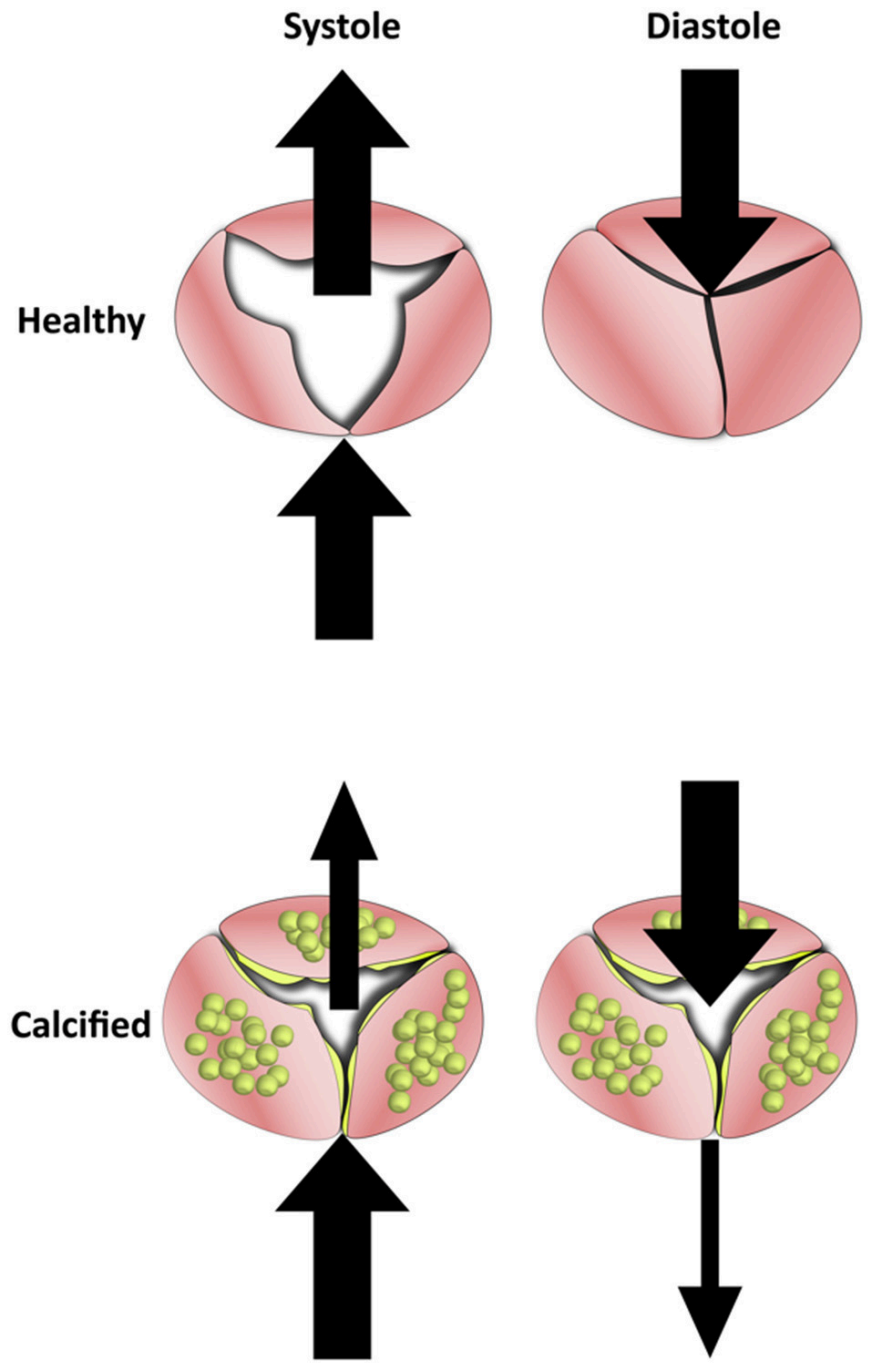
The Effects of calcification on blood flow in the aortic valve. Healthy valve leaflets are able to move according to blood flow which allows them to open completely under systolic forces and coapt under diastolic. In calcified valves, leaflets lose their flexibility and become rigid. This results in valves that can neither open or close fully, which reduces mean ejection fraction and increases regurgitation.

In late stage calcification, the aortic leaflets become thickened with calcific nodules and overproduction of ECM. The changes in the macro geometry of the leaflets significantly affects the ability of the valve to successfully coapt. While healthy valves fully open under systolic pressure and fully coapt under diastolic, the more rigid and thick calcified valves are impeded in either movement. This reduced motion lowers the ejection fraction of the valve while also increasing retrograde leakage into the ventricle (Figure 6).

### Vascular Calcification

Shear stress is required for the proper physiological functioning of the endothelial layer in vessels. Prolonged and continuous pulsatile shear stress ensures that the genes necessary for shielding vessel walls from inflammation are being properly transcribed (83). Low shear stress is responsible for numerous potential causes for calcification. Interestingly, decreases in vascular shear stress may be attributed in part to calcification of the aortic valve, because the aortic valve is the regulator for downstream pressure in the vasculature. When the aortic valve is compromised by calcification, pulse pressure in arteries are markedly lower than in the physiologically healthy system (9, 84). Disruption of the physiological hemodynamics causes noted disruption of the endothelial layer leading to calcification. Changes in the endothelial layer, markedly EndoMT, have been shown to contribute to atherosclerotic plaque calcification (85).

Similar to valvular calcification, vascular calcification itself, brought on by atherosclerotic plaques and other clinical conditions, may reduce WSS, further propagating more mineralization in a vicious cycle. Although it is unknown whether calcification causes changes in shear stress or conversely whether changes in shear stress cause calcification, it has been demonstrated that regions of vasculature with lower shear stresses have more atherosclerosis and calcification, suggesting that some relationship exists (47, 86).

The production of NO is stimulated through constant, pulsatile shear stress. In vascular regions experiencing low shear stress, NO becomes less available, leaving vasculature prone to thrombosis and inflammation. Furthermore, low shear stress downregulates the powerful vasoconstrictive molecule prostacyclin, further threatening the onset of calcification in vessels (46).

Low shear stresses, associated with vasculature downstream of a bifurcation, also put vessels at risk for infiltration of VSMCs from the *tunica media* into the endothelial cell layer of the *intima* (47). Proliferation of VSMCs in the *intima* causes overproduction of fibrillar collagen, which eventually coalesces to form the structure of calcific plaques that calcify over time. Additionally, the increase residence time of blood in contact with the vessel wall associated with lowered shear stress results in the increase of LDL cholesterol uptake by vasculature and the upregulation of reactive oxygen species (ROS). The LDL particles become oxidized, which triggers the recruitment of inflammatory cells in an attempt to rid the vessels of these “foreign” particles. These inflammatory cells are able to easily infiltrate arterial wall due to the low shear stress and high residence time, which is a major contributing factor to the calcification of vasculature (47). As a result of these changes, inflammation and atherosclerosis are found to occur at greater rates in vascular regions distal to bifurcations (47, 48, 87).

Bifurcations themselves are also prone to calcification. The forces experienced at the point of bifurcation differ from those in straight arterial regions, leaving these regions at greater risk for variable blood pressure. Since they experience a lower mean WSS, bifurcations consequently face the risks associated with low shear stress as mentioned earlier. Furthermore, bifurcations frequently encounter reversed flow during systole, increasing the residence of blood in those regions, again contributing to calcification as previously discussed (88).

Other molecular bases for the onset of vascular calcification are plentiful. One of the most significant contributors is osteopontin (OPN), a matrix protein largely responsible for the inhibition of calcification. OPN blocks the growth of calcium crystals throughout the body, which prevents the pathogenic onset of ectopic calcification (8). In an *in vitro* study of bovine smooth muscle cells, exogenous OPN treatment inhibited the spread of calcification (89). Furthermore, mice lacking OPN and Matrix Gla Protein (MGP), an inhibitor of bone morphogenic protein, had greater instances of calcification compared to their healthy mice counterparts (90, 91). Reduction of MGP has also been linked to inducing EndoMT via activation of both elastases and kallikreins resulting in further calcification (92). Interestingly, rats with high levels of calcification were found to have greater concentrations of MGP compared to healthy counterparts (93). This suggests that MGP might have a role in minimizing the propagation of further calcification. Osteoprotegerin (OPG), like OPN and MGP, is also speculated to play a role in vascular calcification. OPG is believed to inhibit RANKL, which is responsible for the maturation of osteoclast progenitors. Mice deficient in osteoprotegerin were found to have calcification of the aorta and renal arteries (94).

Beyond decreases in shear stress, another calcification-related change in the vasculature is the degradation of elastin fibers. Although VSMCs are normally found in an ECM rich in elastin, the rate of synthesis of elastic fibers by adult VSMCs is very low, which is detrimental in diseases involving degradation of elastin (95). The degradation of elastin fibers is highly correlated with calcification in vessels. It has been shown that cells going through EndoMT during calcification secrete elastases that contribute to this degradation due to the reduction in MGP mentioned previously (85). Elastic fibers are crucial in vascular tissues' ability to recoil and respond to normal (pressure-related) stresses (96), and alterations in those stress responses can greatly contribute to vascular calcification. *In vivo* studies in rats have shown that areas of elastin breaks in vessels were significantly correlated with higher levels of calcium depositions (84, 97). Furthermore, the breakdown of elastin fibers in areas with calcification caused decreased arterial compliance, and rats exhibiting greater levels of calcification had greater vascular wall thickness overall (97). As with shear stress and calcification, the causal effect between decreased elasticity and calcification is unknown, but such relationships may indicate that increased stiffness can have an effect on vulnerability to vascular calcification.

While much of this discussion has focused on the calcification of the intimal layer of arteries, the *tunica media* similarly experiences calcification. Medial artery calcification (MAC), also known as Mönnckeberg's arteriosclerosis, leads to the stiffening of the elastic fibers in the smooth muscle layer of the arterial wall. MAC, once thought of as benign, has been recognized to be one of the greatest indicators of cardiovascular death (84, 98). MAC is most commonly associated in patients with chronic kidney disease and type II diabetes mellitus (98).

MAC has similar chemical triggers as with the calcification of the intimal layer, such as overexpression of MGP and the under-expression of OPN. In patients with chronic kidney disease, the greater levels of calcium phosphate found in the blood leads to depositions predominantly into the elastin-rich medial layer. The depositions create calcium phosphate crystals, which triggers the VSMCs to express bone-related genes, such as OPN (99).

In type II diabetes mellitus, hydroxyapatite is the most abundant mineral deposited, leading to MAC (47, 84). Crystallization of hydroxyapatite results in depositions of calcium in the medial layer, leading to the arterial stiffening. Reduced elasticity and reduced arterial compliance not only further propagates vascular calcification, but can also lead to decreased blood flow in diabetic patients. As a result, presence MAC is often the major factor in determining the necessity of amputation of limbs in type II diabetes mellitus (84, 98). MAC may also simply be associated with aging. Due to the effect of aging on reduced elasticity of tissues and general renal insufficiency amongst many other factors, calcification may develop as a consequence of a natural biological process (84).

### Comparison of Calcification Pathology

Vascular and valvular calcification have independent and convoluted disease pathways, even though they do have some similarities in how the diseases progress. Calcification is observed in both appearances to revolve around a disrupted endothelial layer. This initial disruption is similar in both diseases; lower pressure, oscillatory shear is present on the non-coronary valve (the one most often presenting calcification) and in the segments of vasculature most prone to calcify. Mechanical properties of both vascular and valvular tissue change during the progression of the disease, specifically by becoming more rigid and less elastic. Due to changes in the mechanical and cellular environments, NO concentrations are reduced in both presentations of calcification.

While there are many similarities in the calcification of vasculature and valves, there are also significant differences that should not be overlooked (Table 1). In CAVD, the VICs play a large role in manipulating the ECM environment and differentiating into osteogenic cells. These myofibroblast cells are dissimilar to VSMCs, although VSMCs are also not terminally differentiated. Possibly the most noteworthy difference between these calcifications is in the presentation of the calcific nodules in the tissue. In CAVD, mineral deposits create calcific nodules similar to new bone deposition, whereas vascular calcification presents as a lipid-laden plaque.

**Table 1:** Overview of Similarities and Differences of Calcification in Valves and Vasculature.

**Similarities**	**Differences**
Occurs in areas of low and oscillatory shear flow	Presents in different morphology (plaque vs. bone-like nodules)
Specific geometry dictates hemodynamic forces	Clinically different manifestations (atherosclerosis vs. CAVD)
Calcify due in part to endothelial lining disruption	Different interstitial cells maintaining homeostasis

## Variation in Disease Prediction and Treatment

### In CAVD

Valvular stenosis and calcification can be predicted through their risk factors of diabetes, smoking, hypertension, abundance of lipids in the blood system, and various metabolic syndromes (100). Further indicators of disease are smoking, higher body mass index, and high cholesterol (2). Unlike some heart conditions, people of male gender are more prone to stenosis and calcification than females (101). The most correlated risk factor for CAVD, however, is aging. While it is now accepted that calcification is not due to passive degradation throughout patient lifetime, aging is still the best predictor of disease onset. Over time, the amount of calcium and other minerals accumulating within the valve increases, which creates a propensity toward calcification (102).

Currently, the only treatment for patients with a highly calcified valve is surgical replacement. No non-surgical treatment exists, although statins (a common treatment for atherosclerosis) were previously tested on CAVD patients but showed no decrease in disease progression. Recent findings have also suggested that vitamin K treatment in patients with mild and moderate stenosis can reduce the progression of aortic calcification, although due to the limited patient population this study did not show changes in valve functionality (103). Due to complications with surgery and relatively high event-free survival statistics in mild cases, patients who have aortic calcification but are asymptomatic are usually recommended to withhold from surgery (50). In contrast, patients with severe calcification are frequently referred to get immediate treatment as the event-free survival rate at 1 year is only 60%, and drops to 47 and 20% at 2 and 4 years, respectively (50, 104).

Options for replacement are either mechanical valves or biological valves. Mechanical valves used to make up the majority of implanted valves, but have been surpassed in use by biological valves (105, 106). Mechanical valves require daily anti-coagulant medication due to the immune response elicited at the metal-blood interface. Biological valves can either be cryopreserved human explants or chemically fixed animal tissue. Due to a lack of organ donors, bioprosthetic implants are mostly made from porcine or bovine tissue. Bioprosthetic valves have an average life span of 15 years before failure due to structural deterioration, requiring reoperation to replace the valve again (107). Patient outcomes at reoperation are poorer due to increased age and poorer overall health of most patients. The major recent advancement in the field of bioprosthetic valves has been the introduction of trans-catheter heart valve replacement (108, 109). This system allows for the delivery of a biological valve via intravenous balloon catheterization. Studies of the long-term performance of trans-catheter valves show this implementation method improves outcomes for high risk patients by circumventing the need for open heart surgery while still maintaining the implant integrity (110, 111). However, this treatment is currently only used on those not fit for surgery and remains a small fraction of total replacement surgeries.

The newest research in heart valve replacement is tissue-engineered heart valves (TEHVs) which are not currently commercially available but show promise in initial studies and animal models (112–114). The intention of TEHVs is to have a replacement valves that adapts and grows with patients, as that is one of the main limitations of bioprosthetic valves, and is a necessary adaptation for pediatric patients (115). Pediatric recipients of artificial heart valves require replacement surgeries more often than their adult counterparts due to their growing bodies and their higher susceptibility to prosthetic valve complications and failure (108, 116). Indeed, young age remains the primary risk factor for early failure of bioprosthetic valves (116, 117). Thus, the emergence of TEHVs would be a valuable addition to the range of commercially available valve implants. TEHVs are generally created through a combination of ECM scaffolding populated with cells. These cells can either be added during the TEHV creation, or a decellularized scaffold can be implanted and recellularized via circulating cells in the blood stream (107, 118). Early clinical attempts at TEHVs, however, have suffered from fibrosis-like failure due to the continuous activation of the reseeded cells causing rampant collagen formation (115). The functional performance of the TEHVs has also been found to decrease over time as adverse remodeling of the valve prohibits successful coaptation, although initial studies from Emmert et al. have demonstrated that changing the initial geometry of TEHVs can reduce undesirable remodeling (119). While progress in this field is underway, performance issues such as these have prevented TEHVs from becoming a commercial reality as of yet (120).

### In Atherosclerosis

A prediction of vascular calcification is largely contingent upon the preexisting conditions a patient may have. As stated earlier, vascular calcification is very rarely found in isolation. Patients who are diabetic, post-menopausal, experience renal failure, or suffer from other conditions that downregulate calcium inhibitors should be cognizant of the greater risk for vascular calcification they face (84, 97). Similarly, individuals already experiencing aortic calcification and/or early-stage atherosclerosis need to be consistently monitored for worsening calcification of peripheral vasculature.

The treatment and regulation of concomitant conditions associated with vascular calcification may have an ability to control vascular calcification and prevent the worsening of symptoms. When paired with advanced kidney disease, treatments focus on reducing circulating calcium and phosphate levels (121). When vascular calcification is seen with atherosclerosis, statins have are used to reduce the rate of disease progression (122). While this treatment does not cause diseased tissue to revert to normal, it is effective in pausing the enlargement of atherosclerotic plaques (123).

Treatment options specific to combatting vascular calcification are not a current clinical prospect, although treatments are currently under investigation in animal studies. Vitamin K, specifically vitamin K2, has been found to be inversely related to severe aortic calcification (124). Proteins dependent on vitamin K have been studied to potentially inhibit vascular calcification, such as MGP. Availability of vitamin K is a major factor for the activation of these proteins. Increasing intake of this vitamin therefore has the potential to prevent the progression of calcification (124). In a more recent study, post-menopausal women, who are at greater risk for vascular calcification, were shown to have a decreased risk of coronary calcification with increased vitamin K2 intake (125). Less studied options include statins, bisphosphonates, TNAP inhibitors, and non-steroidal anti-inflammatory drugs (121). While these treatments must be investigated further, preliminary studies have found results in the ability to control either plaque or calcification in non-vascular regions, providing hope that applications in vasculature may exist (9).

Surgical treatments are an option for late-stage vascular calcification. In late-stage calcification, the diameter of the carotid artery can significantly reduce causing higher odds of fatal strokes. Carotid endarterectomies are common practice when patient's carotid artery is severely calcified (>70% reduction in diameter) to remove material from the artery. For these patients, endarterectomies can reduce their 5-year risk of stroke occurrence from 12 to 6% (126). Recent studies have found that a modified version of this surgery, eversion carotid endarterectomy, might have better peri-operative outcomes but performs similarly for post-operative outcomes (127).

### Comparison of Diagnosis and Treatment

Predicting onset of either form of clinical calcification remains an inexact science. CAVD is predicted predominantly via advanced age while vascular calcification is monitored when a known disease initiator is already present. While there are no non-surgical treatments for CAVD, the progression of vascular calcification can be hindered by treating the preceding disease that instigated the calcification. Statins are lipid-lowering drugs that have been shown to be effective at reducing the progression of atherosclerotic plaques. Even with the similarities between CAVD and vascular calcification, when statins were used to treat CAVD there was no evidence that they halted calcification. This is likely due to the extreme differences in their presentation of calcification. The plaques formed in atherosclerosis are lipid-rich deposits whereas CAVD is a more mineral and bone-like morphology. Correspondingly, the lipid-lowering statins had little effect in CAVD. Based on the research presented in this review, treatment options targeting CAVD should be investigated using either aspects of the disease that are unique to calcified valves, or bona fide commonalities between the two diseases. For instance, both a reduction in NO production and the disruption of the endothelial layer are shown to be initiators of severe valvular and vascular calcification. Targeting these pathways may lead to treatments that would be functional for both diseases.

## Conclusions

Cardiovascular calcification is pervasive throughout the older population of adults in the United States. As our national demographic skews toward having longer expected lifespans, calcification will only become a more dominate issue. Thus, important steps need to be taken in researching treatments and interventions. As found in both in the aortic valve and in vasculature, calcification modifies the physiological environment of tissue beyond repair. While they are often studied separately, the processes of valvular and vascular calcification share many similarities that might be worth exploring. Specifically, the oscillatory shear stresses at both the non-coronary leaflet of the aortic valve and downstream of bifurcations in vasculature seem to play a vital part in the initiation and progression of calcification. Further, the role of disrupted endothelium layers are significant in both areas of calcification. They also share NO inhibition which destabilizes the interior cells (VICs and VSMCs respectively) and leads to calcification. With these similarities, the differences between valvular and vascular calcification must be appreciated to study them properly. The mechanical environment of the AV is cyclic and multidimensional, whereas vasculature experiences more steady flows. Further, the differences between their interior cells, their phenotypes, regulation of ECM and mechano-sensitivity likely plays a large part in the variations observed between these diseases. In summary, valvular and vascular calcification requires further study to explore proper treatment options. It may be beneficial to look at these seemingly incongruous diseases jointly to learn more about the initiation, progression and inhibition of calcification.

## Author Contributions

MG wrote the valvular sections of this paper, the summaries, the abstract, introduction, and conclusion, and conceptualized and made figures. RL wrote the vascular sections and made figures. KG-A contributed to the formation and editing of this manuscript.

### Conflict of Interest Statement

The authors declare that the research was conducted in the absence of any commercial or financial relationships that could be construed as a potential conflict of interest.
